# Evaluation of factors related to Anaesthesia-induced Lens opacity in experimental mice

**DOI:** 10.1186/s42826-019-0031-z

**Published:** 2020-01-07

**Authors:** Hun Lee, Hong Kyung Kim, Hae Sol Shin, Soo Jung Han, Sangchul Yoon, Je Kyung Seong, Kyoung Yul Seo

**Affiliations:** 1 Department of Ophthalmology, Asan Medical Center, University of Ulsan College of Medicine, Seoul, South Korea; 2 Korea Mouse Sensory Phenotyping Center, Yonsei University College of Medicine, Seoul, South Korea; 3 The Institute of Vision Research, Department of Ophthalmology, Yonsei University College of Medicine, Seoul, South Korea; 4 Laboratory of Developmental Biology and Genomics, BK21 Program Plus for Advanced Veterinary Science, and Research Institute for Veterinary Science, College of Veterinary Medicine, Seoul National University, Seoul, South Korea; 5 Korea Mouse Phenotyping Center, Seoul, South Korea

**Keywords:** Lens opacity, Anaesthetic dose, Ocular surface dryness, Infrared light, C57BL/6 N mice

## Abstract

To investigate conditions that cause temporal lens opacity, we tested chemical and physical factors, such as anaesthesia dose, ocular surface dryness, and infrared (IR) light exposure in anaesthetised C57BL/6 N mice. Mice were anaesthetised with a low (80%; tiletamine/zolazepam 32 mg/kg and xylazine 8 mg/kg, intraperitoneal injection) or high (120%; 48 mg/kg and 12 mg/kg) dose of anaesthetic and examined every 5 min from 10 to 30 min after anaesthesia was induced. Lens opacity levels were assessed and graded (1–6) using the standard classification system. Regardless of the anaesthetic dose, lens opacity grade was 1–2 in moisturised eyes with application of 0.5% carboxymethylcellulose, and 5–6 in dry ocular surface conditions. Lens opacity in mice with high-dose anaesthetic in the dry ocular surface condition was not different from that of mice with low-dose anaesthetic. Lens opacity grade 1–2 was noted in eyes in the wet ocular surface condition, regardless of IR light exposure. During IR light exposure in eyes in the dry ocular surface condition, lens opacity (grade 6) in mice with high-dose anaesthetic was not different from that (grade 6) in mice with low-dose anaesthetic. We demonstrated that ocular surface dryness might be a relevant factor for the formation and progression of lens opacity in anesthetized C57BL/6 N mice. Anaesthesia dose and IR light exposure did not strongly influence lens opacity formation. Furthermore, eyes with corneal dryness-induced lens opacity recovered to normal status without additional intervention.

## Introduction

During observation of the posterior segment of the eye, transparency of the ocular media is essential, because opaque medium, especially lens opacity, significantly affects accuracy and results of measurement. Lens opacity is induced by genetic, developmental, and environmental cues. It is sometimes difficult to discriminate short-term lens opacity from cataracts that permanently cloud the lens and impair vision. Interestingly, 90 genes were annotated as associating with abnormal lens morphology in the International Mouse Phenotyping Consortium database, whereas 51 genes were similarly annotated by the German Mouse Clinic.

Because many genes have been annotated as associating with lens abnormalities, there is a need to determine the various factors causing them. It has been reported that various chemical and physical factors, including drugs, anaesthetics, oxygen supply, calcium, pH stress, and dehydration, affect the formation of lens opacity in mice and rat [1–4]. Further, the duration of the scanning procedure and dose of anaesthesia should be minimised, and body temperature should be maintained, to obtain reproducible outcomes in anaesthetised mice [1–4]. Although there is a consensus that anaesthetic dose, dehydration, and temperature are the most important factors affecting the formation of lens opacity, a precise explanation for the interrelationship of these factors remains ambiguous [1, 5, 6].

**Fig. 1:**
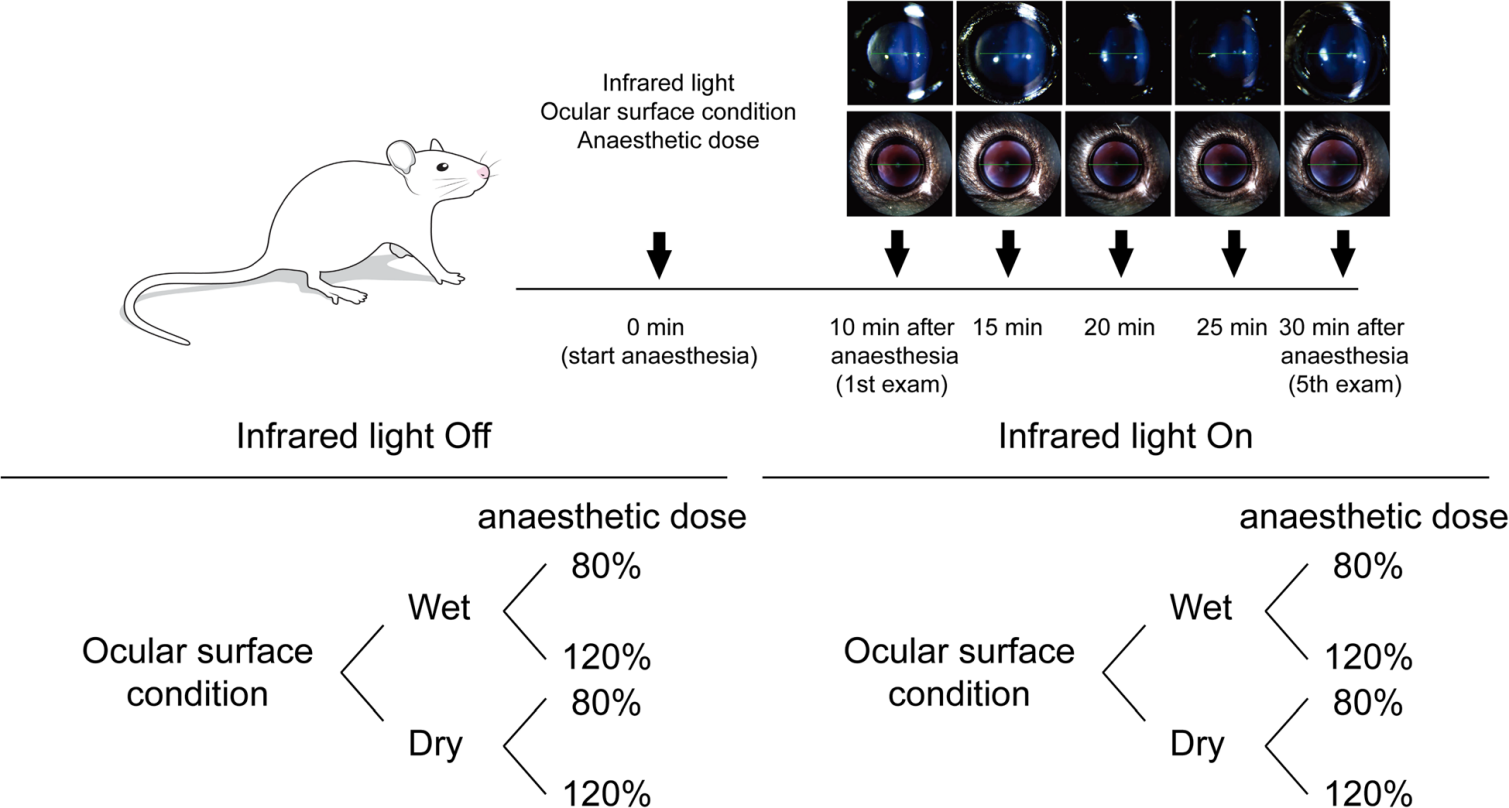
Schematic representation of experimental design

Therefore, in the present study, we aimed to investigate the effect of anaesthetic dose, ocular surface dryness, and the presence or absence of infrared (IR) light on the formation of lens opacity in C57BL/6 N mice. We documented the time course of lens opacity formation by using the Micron Image-Guided Spectral-domain optical coherence tomography (OCT) system (Phoenix Research Labs, Pleasanton, CA, USA) and slit-lamp biomicroscopy incorporated into the OCT device (Phoenix Research Labs) (Fig. 1). We also performed quantitative analyses among various conditional groups after establishing standardised lens opacity grading.

## Materials and methods

### Animal

Male C57BL/6 N mice, aged 14–16 weeks, were supplied by Jackson Laboratories (Bar Harbor, ME, USA). Mice were maintained in an experimental animal facility under specific pathogen-free conditions at Yonsei University College of Medicine (Seoul, South Korea). Mice were housed under a 12-h light/dark schedule (lights on at 6 am, off at 6 pm) with ad libitum access to autoclaved food and water; they were treated humanely and with regard for the minimisation of suffering.

### Anaesthesia

Mice were anaesthetised with a low (80%; tiletamine/zolazepam 32 mg/kg and xylazine 8 mg/kg, intraperitoneal injection) or high (120%; tiletamine/zolazepam 48 mg/kg and xylazine 12 mg/kg, intraperitoneal injection) dose of anaesthetic and examined every 5 min from 10 to 30 min after anaesthesia was induced. Pupils were dilated with 0.5% tropicamide/0.5% phenylephrine mixed eye drop (Mydrin-P, Santen, Osaka, Japan) immediately after anaesthesia. Then, eyes with clear ocular media, as defined by OCT and slit-lamp biomicroscopy, were assessed. Lens opacity were imaged by the Micron Image Guided Spectral-domain OCT system after applying special instrument for examining the anterior segment and slit-lamp biomicroscopy which is incorporated with OCT system.

### Experimental design

**Fig. 2:**
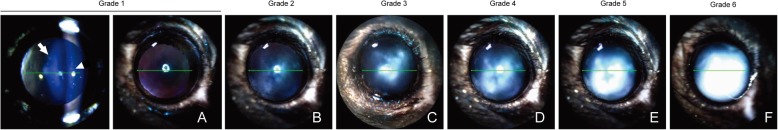
Visual classification system of progressive lens opacity. **a** Transparent lens (grade = 1); **b** very mild lens opacity located in the centre of the anterior lens (grade = 2); **c** moderate lens opacity located in the central region (grade = 3); **d** moderate lens opacity located in both central and peripheral regions (grade = 4); **e** nearly complete opacification in the pupil area (grade = 5); **f** complete opacification (grade = 6). arrowhead = cornea, arrow = lens opacity

All experimental procedures were completed in the Department of Laboratory Animal Resources of Yonsei Biomedical Research Institute and Yonsei University College of Medicine. First, we modified the previous simple classification system described by Bermudez et al. to develop our own lens opacity severity classification system [1]. Fig. 2 shows the system of visual classification of six grades of lens opacity from grade 1 (clear lens) to grade 6 (very severe opacity).

Eight groups of mice (at least three mice per group) were used to investigate the effect of three factors on the formation of lens opacity, including anaesthetic dose, ocular surface dryness, and IR light exposure (Fig. 1). Anaesthetised mice were examined every 5 min from 10 to 30 min after anaesthesia was induced.

Briefly, to test whether ocular surface dryness affects lens opacity in the absence of IR light exposure (only exposed during OCT examinations), we did not apply preservative-free artificial tears for a total of 30 min. Conversely, the corneas were kept moist with application of preservative-free 0.5% carboxymethylcellulose every 1 min after anaesthesia was induced with a low or high dose of anaesthetic. The same experiments were performed in the presence of IR light exposure (continuous exposure from 10 to 30 min after anaesthesia). Next, to examine whether anaesthetic dose affects lens opacity, mice were anaesthetised with a high or low dose of anaesthetic and examined without IR light exposure, every 5 min from 10 to 30 min after anaesthesia was induced in each ocular surface condition. The same experiments were performed in the presence of IR light exposure.

Finally, we explored the reversibility of lens opacity. Lens opacity of grade 5–6 was induced by ocular surface dryness after anaesthesia was induced with a low dose of anaesthetic (*n* = 5). Then, lens transparency was assessed at 0, 1, 2, 4, and 6 h after induction of lens opacity of grade 5–6. Additionally, moderate lens opacity was induced by IR light exposure for 40 min in mice without anaesthesia (*n* = 5). In the same manner, lens transparency was assessed at 0, 1, 2, 4, and 6 h after induction of lens opacity of grade 3–4.

### Statistical analysis

Data are reported as the mean ± standard deviation. The Mann-Whitney U test and Wilcoxon signed-rank test were performed for comparison of data. Statistical analyses were performed using GraphPad PRISM software (GraphPad Software, Inc., La Jolla, CA, USA). Differences were considered to be statistically significant at *p*-values of < 0.05.

### Ethics statement

This study was conducted in strict accordance with and adherence to the relevant national and international guidelines regarding animal handling as mandated by the Institutional Animal Care and Use Committee (IACUC) of the Yonsei University Health System (Seoul, Korea). The committee has reviewed and approved the animal study protocol (#2011–0137). All experimental protocols were conducted in accordance with the tenets of the Declaration of Helsinki and the Association for Research in Vision and Ophthalmology (ARVO) Statement on the Use of Animals in Ophthalmic and Vision Research.

## Results

### Effect of anaesthetic dose on lens opacity formation in wet ocular surface condition

To evaluate degrees of lens opacities resulting from various factors, we collected sample pictures from all image data and established classification standards consisting of six grades of lens opacity. Lens opacity initially formed in the centre of the anterior lens and progressively expanded towards the periphery (Fig. 2). A modified visual classification system of progressive lens opacity was used to assess the severity of lens opacity on the basis of six grades of lens opacity, from grade 1 (clear lens) to grade 6 (very severe opacity), all of which were based on images obtained from OCT and slit-lamp biomicroscopy examinations.

**Fig. 3:**
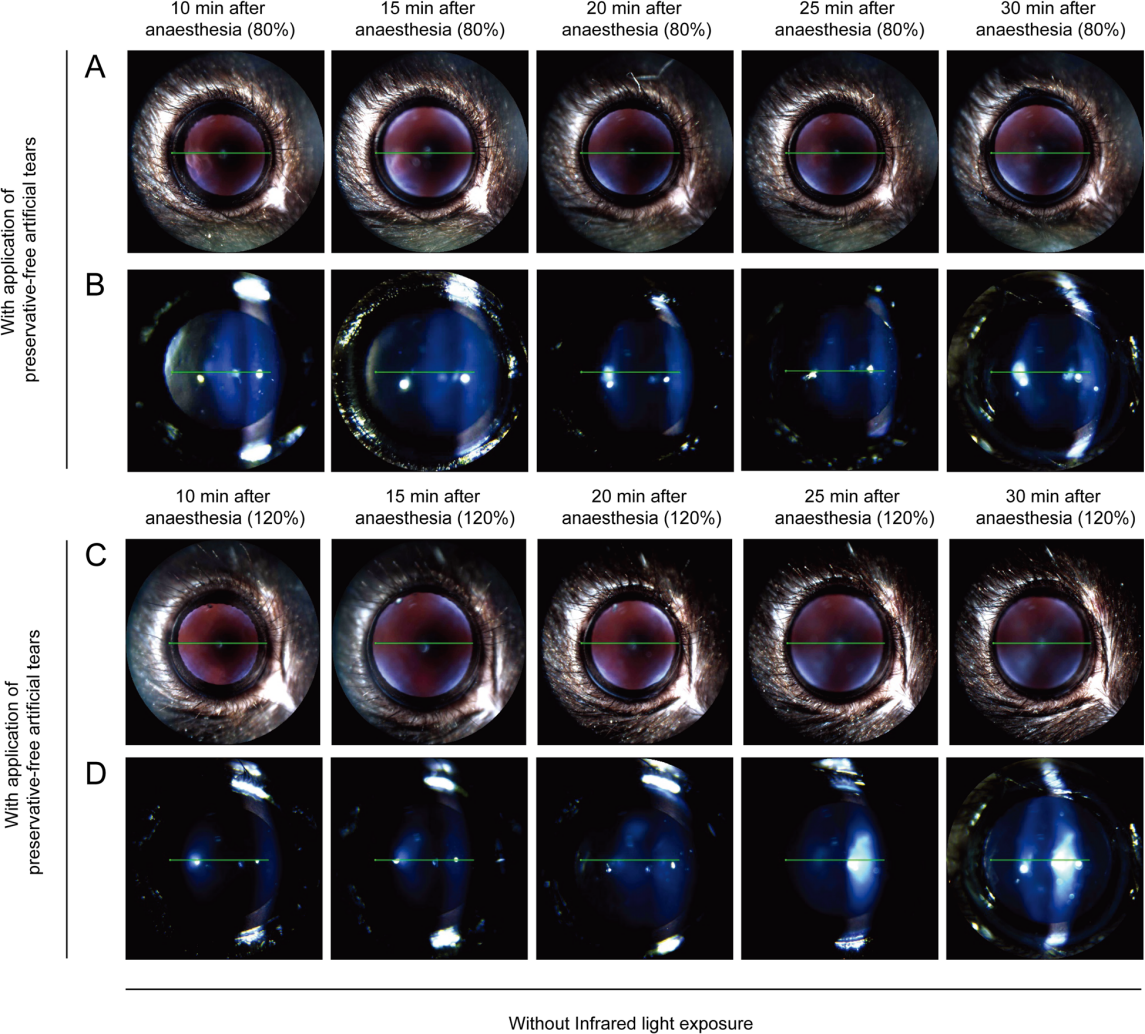
Comparison of lens opacity induced by different doses of anaesthesia with regular application of preservative-free artificial tears in anaesthetised mice. Mice were anaesthetised with a low (80%) or high (120%) dose of anaesthetic and examined every 5 min from 10 to 30 min after anaesthesia. **a** and **b** anaesthetised with tiletamine/zolazepam (32 mg/kg, intraperitoneal injection) and xylazine (8 mg/kg, intraperitoneal injection) (80% setting) without infrared light exposure. **c** and **d** anaesthetised with tiletamine/zolazepam (48 mg/kg, intraperitoneal injection) and xylazine (12 mg/kg, intraperitoneal injection) (120% setting) without infrared light exposure

Next, we investigated whether anaesthetic dose affected the formation of lens opacity. Mice were separated into two groups and administered a low (80%) or high (120%) dose of anaesthetic, respectively. First, without IR light (exposure solely during OCT examinations), mice were examined every 5 min from 10 to 30 min after anaesthesia was induced with application of preservative-free 0.5% carboxymethylcellulose (Refresh Plus; Allergan Inc., Irvine, CA, USA) every 1 min. The grade of lens opacity in eyes with a high dose of anaesthetic was similar (grade 1–2) to that of eyes with a low dose of anaesthetic (Fig. 3). Lens opacity of grade 1–2 was revealed in moisturised eyes with both low and high doses of anaesthetic during 20 min of imaging (Fig. 3).

### Effect of anaesthetic dose on lens opacity formation in dry ocular surface condition

**Fig. 4:**
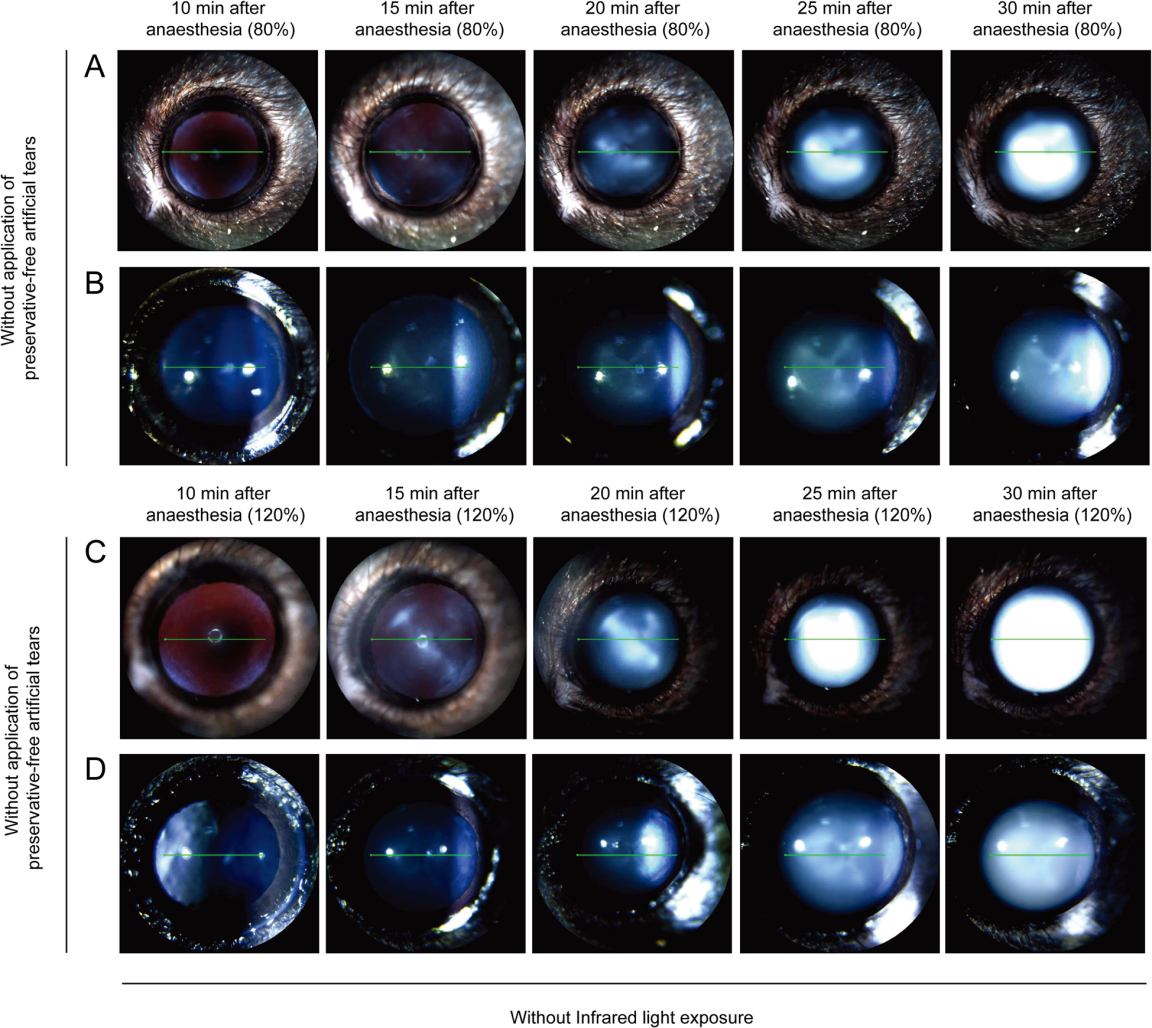
Comparison of lens opacity induced by ocular surface dryness in mice anaesthetised with different doses of anaesthetic. Mice were anaesthetised with a low (80%) or high (120%) dose of anaesthetic and examined every 5 min from 10 to 30 min after anaesthesia without application of preservative-free artificial tears and infrared light exposure. **a** and **b** anaesthetised with tiletamine/zolazepam (32 mg/kg, intraperitoneal injection) and xylazine (8 mg/kg, intraperitoneal injection) (80% setting) without application of preservative-free artificial tears. **c** and **d** anaesthetised with tiletamine/zolazepam (48 mg/kg, intraperitoneal injection) and xylazine (12 mg/kg, intraperitoneal injection) (120% setting) without application of preservative-free artificial tears

In the absence of IR light exposure, mice received a low or high dose of anaesthetic and were examined every 5 min from 10 to 30 min after anaesthesia was induced, without application of preservative-free 0.5% carboxymethylcellulose. Compared with mice that underwent continued application of moisture to the eye, a significant higher degree of lens opacity (grade 2–4; four of four mice) was observed in the dry ocular surface condition at 20 min after anaesthesia, despite the application of a low dose of anaesthetic (*p* < 0.05) (Fig. 3a and 4a). Lens opacity of grade 5–6 appeared in four of four mice at 30 min after anaesthesia. In the dry ocular surface condition, lens opacity in mice with a high dose of anaesthetic was not different from that in mice with a low dose of anaesthetic (Fig. 4). Regardless of anaesthetic dose, lens opacity of grade 5–6 appeared at 30 min after anaesthesia (Fig. 4).

### Effect of ocular surface dryness on the formation of lens opacity

To determine whether lens opacity is influenced by ocular surface dryness, we monitored mouse eyes and obtained images in the absence or presence of moisture. Mice were anaesthetised with a low dose of anaesthetic and examined bilaterally every 5 min from 10 to 30 min after anaesthesia was induced. The right eye was kept moist with regular application of preservative-free artificial tears every 1 min, and the left eye was kept dry throughout the experiment. Lens opacity of grade 1–2 appeared in moisturised eyes during 20 min of imaging (Fig. 3a and b), whereas more severe lens opacity (grade 5–6; four of four mice) was present in eyes in the dry ocular surface condition (Fig. 4a and b). Cloudy lens began to be formed at 20 min after anaesthesia and became progressively thicker and larger.

Next, mice were anaesthetised with a high dose of anaesthetic and examined bilaterally every 5 min from 10 to 30 min after anaesthesia. The right eye was kept moist with regular application of preservative-free artificial tears, and the left eye was kept dry throughout the experiment. Compared with mice that received continued application of moisture to the eye (grade 1–2; Fig. 3c and d), a significantly higher degree of lens opacity (grade 5–6; Fig. 4c and d) occurred in four of four mice in the dry ocular surface condition at 30 min after anaesthesia (*p* < 0.05).

### Effect of anaesthetic dose on lens opacity formation with infrared light exposure

**Fig. 5:**
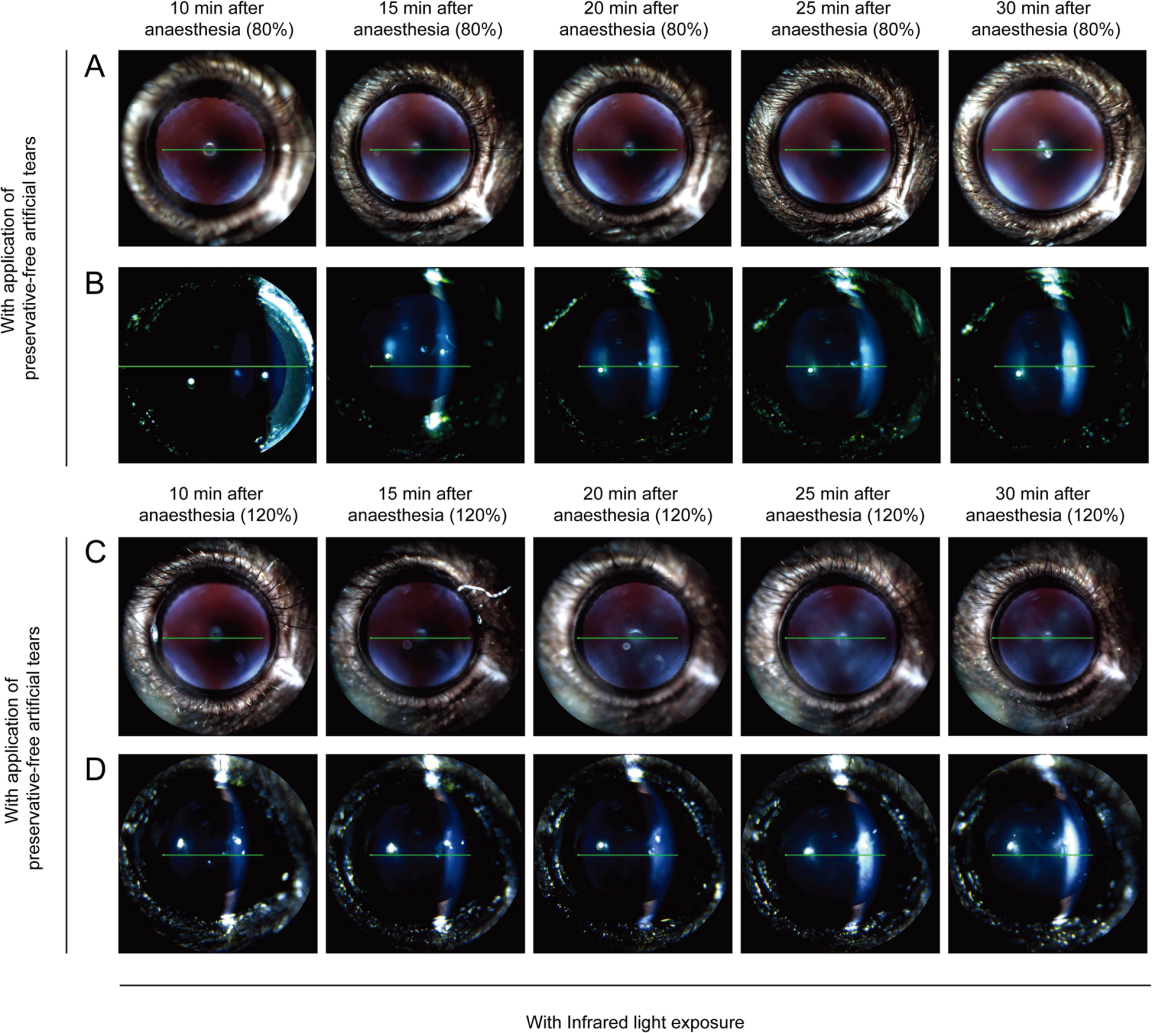
Comparison of lens opacity induced by different doses of anaesthesia with regular application of preservative-free artificial tears and infrared light exposure. Mice were anaesthetised with a low (80%) or high (120%) dose of anaesthetic and examined every 5 min from 10 to 30 min after anaesthesia with infrared light exposure. **a** and **b** anaesthetised with tiletamine/zolazepam (32 mg/kg, intraperitoneal injection) and xylazine (8 mg/kg, intraperitoneal injection) (80% setting). **c** and **d** anaesthetised with tiletamine/zolazepam (48 mg/kg, intraperitoneal injection) and xylazine (12 mg/kg, intraperitoneal injection) (120% setting)

In the presence of IR exposure, mice were examined every 5 min from 10 to 30 min after anaesthesia in the wet ocular surface condition. The grade of lens opacity in eyes with a high dose of anaesthetic was similar to that of eyes with a low dose of anaesthetic (Fig. 5). Moreover, IR light exposure in the wet ocular surface condition did not increase lens opacity, relative to the wet ocular surface condition without IR light exposure (Figs. 3 and 5).

**Fig. 6:**
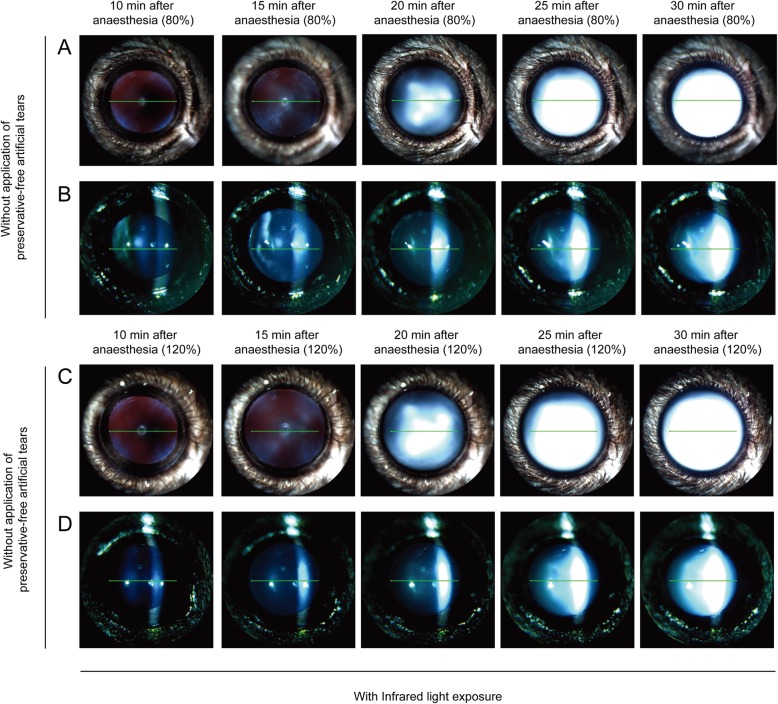
Comparison of lens opacity induced by ocular surface dryness in anaesthetised mice of different doses of anaesthetic with infrared light exposure. Mice were anaesthetised with a low (80%) or high (120%) dose of anaesthetic and examined every 5 min from 10 to 30 min after anaesthesia without application of preservative-free artificial tears. **a** and **b** anaesthetised with tiletamine/zolazepam (32 mg/kg, intraperitoneal injection) and xylazine (8 mg/kg, intraperitoneal injection) (80% setting) without application of preservative-free artificial tears. **c** and **d** anaesthetised with tiletamine/zolazepam (48 mg/kg, intraperitoneal injection) and xylazine (12 mg/kg, intraperitoneal injection) (120% setting) without application of preservative-free artificial tears

Next, with IR light exposure, mice were examined every 5 min from 10 to 30 min after anaesthesia with a low dose of anaesthetic in the dry ocular surface condition (Fig. 6). Compared with mice that received continued application of moisture to the eye, a significantly higher degree of lens opacity (grade 6; three of three mice) occurred in the dry ocular surface condition at 30 min after anaesthesia (Figs. 5 and 6). Lens opacity of grade 3–5 began to be observed at 20 min after anaesthesia in three of three mice (Fig. 6a and b).

During IR light exposure, in mice with a high dose of anaesthetic in the dry ocular surface condition, lens opacity of grade 4–5 (three of three mice) began to be observed at 20 min after anaesthesia; lens opacity of grade 6 (three of three mice) appeared at 30 min after anaesthesia (Fig. 6c and d). During IR light exposure in the dry ocular surface condition, lens opacity in mice with a high dose of anaesthetic did not differ from that in mice with a low dose of anaesthetic (Fig. 6).

Table 1 and demonstrate overall results for lens opacity grade according to anaesthetic dose, ocular surface condition and IR light exposure in anaesthetised C57BL/6 N mice.

**Table 1:** Results for grade of lens opacity according to anaesthetic dose and ocular surface condition in the absence or presence of infrared light exposure in anaesthetised C57BL/6 N mice

	Without infrared light exposure				With infrared light exposure			
	80% anaesthetic dose		120% anaesthetic dose		80% anaesthetic dose		120% anaesthetic dose	
	Wet ocular surface (*n* = 4)	Dry ocular surface (*n* = 4)	Wet ocular surface (*n* = 5)	Dry ocular surface (*n* = 4)	Wet ocular surface (*n* = 3)	Dry ocular surface (*n* = 3)	Wet ocular surface (*n* = 3)	Dry ocular surface (*n* = 3)
10 min after anaesthesia	1.00 ± 0.00	1.00 ± 0.00	1.00 ± 0.00	1.00 ± 0.00	1.00 ± 0.00	1.00 ± 0.00	1.00 ± 0.00	1.00 ± 0.00
15 min after anaesthesia	1.25 ± 0.43	1.25 ± 0.43	1.00 ± 0.00	1.25 ± 0.43	1.00 ± 0.00	1.33 ± 0.47	1.00 ± 0.00	1.00 ± 0.00
20 min after anaesthesia	1.25 ± 0.43	3.25 ± 0.83*	1.00 ± 0.00	3.50 ± 1.12*	1.00 ± 0.00	4.00 ± 0.82*	1.00 ± 0.00	4.66 ± 0.47*
25 min after anaesthesia	1.25 ± 0.43	4.50 ± 0.87*	1.20 ± 0.40	5.25 ± 0.43*	1.00 ± 0.00	6.00 ± 0.00*	1.33 ± 0.47	6.00 ± 0.00*
30 min after anaesthesia	1.25 ± 0.43	5.75 ± 0.43*	1.20 ± 0.40	5.75 ± 0.43*	1.00 ± 0.00	6.00 ± 0.00*	1.33 ± 0.47	6.00 ± 0.00*

Results are expressed as mean ± standard deviation

*p < 0.05 when compared with wet ocular surface in each condition

### Temporal restoration of lens opacity to the normal status

**Fig. 7:**
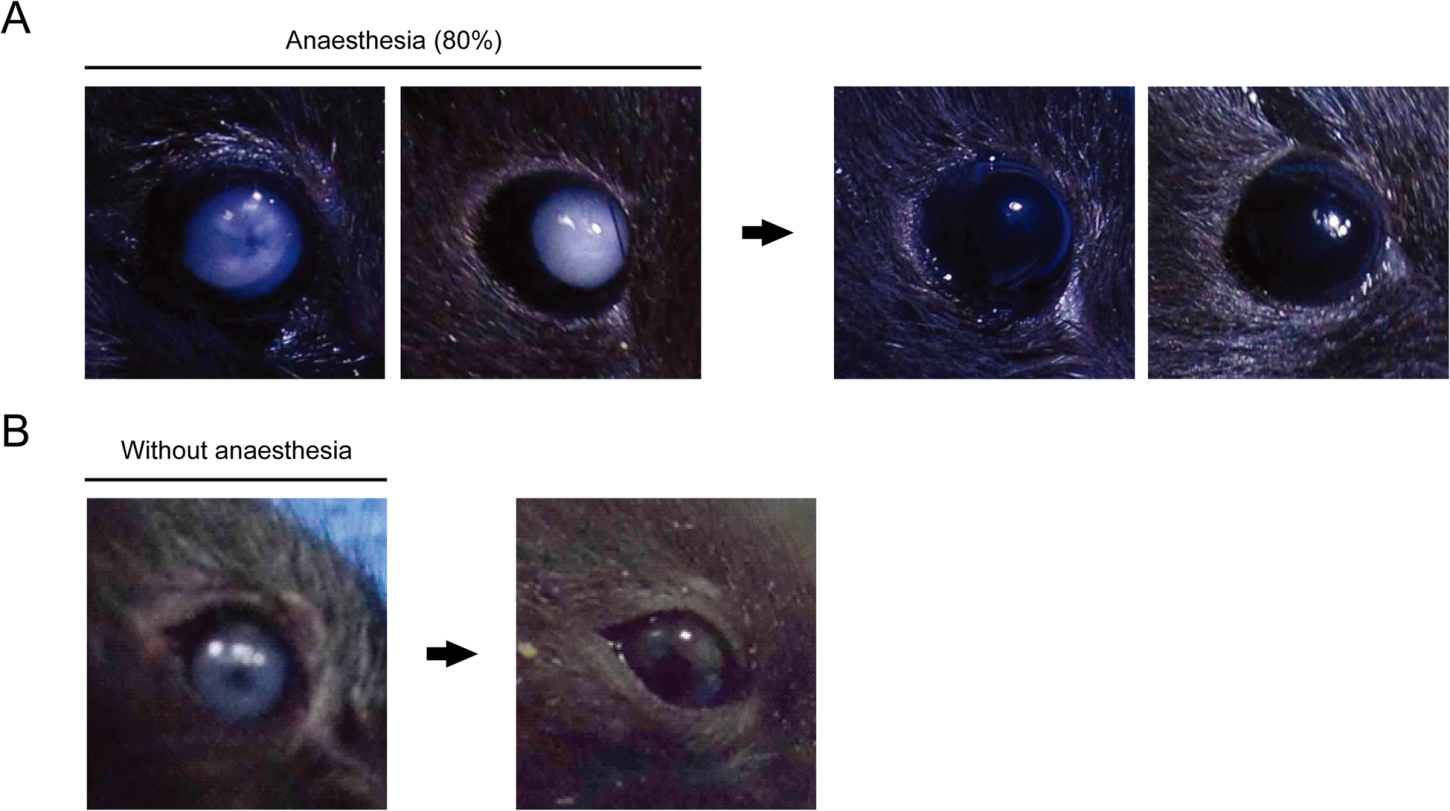
Examples of the reversibility of lens opacity. **a** In anaesthetised mice (80% setting), lens opacity of grade 5–6 induced by ocular surface dryness began to regain transparency at 1 h and showed complete reversion to grade 1 at 6 h. **b** In non-anaesthetised mice, lens opacity of grade 3–4 induced by infrared light exposure of 40 min in wet ocular surface condition began to regain transparency at 1 h and showed complete reversion to grade 1 at 6 h

We tested whether lens opacity resulting from ocular surface dryness could recover to normal status. One hundred percent (5/5) of eyes with lens opacity of grade 5–6 induced by ocular surface dryness began to regain transparency at 1 h; all lens opacity was completely reversed to grade 1 by 6 h (Fig. 7a). These data suggested that lens opacity in the dry ocular surface condition was temporary and did not lead to an irreversible type of cataract. Finally, we tested whether lens opacity resulting from IR light exposure alone without anaesthesia could recover to normal status. One hundred percent (5/5) of eyes with lens opacity of grade 3–4 induced by IR light exposure for 40 min began to regain transparency at 1 h; opacity was completely reversed to grade 1 by 6 h (Fig. 7b).

## Discussion

In the present study, we investigated conditions which cause formation of lens opacity in anaesthetised C57BL/6 N mice using an OCT system and slit-lamp biomicroscopy incorporated within the OCT system. The results demonstrated that ocular surface dryness might contribute to the formation and progression of lens opacity in anaesthetised mice. Furthermore, the formation of lens opacity induced by ocular surface dryness was a reversible phenomenon.

Among many factors, anaesthetic drug, dehydration of the ocular surface, and temperature are involved in the formation of lens opacity [1, 2, 4]. Indeed, dehydration of the ocular surface has been regarded as an important factor in the formation of lens opacity in anaesthetised mice. Thus, there is a need for frequent irrigation with balanced salt solutions or application of a methylcellulose ophthalmic demulcent to prevent the formation of lens opacity during mouse eye experiments [5, 7, 8]. Consistent with the results of previous studies, we showed that dehydration of the ocular surface could be a crucial factor for the formation and progression of lens opacity in anaesthetised C57BL/6 N mice. Notably, it has been speculated that fluid homeostasis, primarily water content and ion concentrations in the anterior segment, have important implications for the formation of lens opacity [9–12]. One recent investigation of the influence of NaCl concentration on lens transparency in anaesthetised mice showed that hypertonic solutions prepared as eye drops can alter the lens transparency of anaesthetised mice much faster and more dramatically than corresponding hypotonic solutions [6]. Although our study did not investigate the effects of different osmotic stress on the formation of lens opacity, the ocular surface dryness assessed here may cause disturbances in osmotic stress and moisture content/ion concentration, ultimately resulting in the formation of lens opacity.

Many anaesthetic drugs are known to inhibit the natural blink reflex and to retract the eyelids, consequently disrupting tear films [2–4, 13]. Thus, anaesthetic drugs themselves are expected to contribute to the formation of lens opacity by causing dehydration of the ocular surface. In this study, we investigated the influence of different concentrations (80% versus 120%) of anaesthetic drug on the formation of lens opacity. In the wet ocular surface condition, a high dose of anaesthetic drug induced a nearly equivalent level of lens opacity to that induced by a low dose of anaesthetic drug. In the same manner, in the dry ocular surface condition, the formation of lens opacity was similar for both concentrations of anaesthetic drug. Our current results confirm that ocular surface dryness facilitates the formation and progression of lens opacity, regardless of the concentration of anaesthetic drug.

We also investigated the effect of IR light exposure on the formation of lens opacity. In both wet and dry ocular surface conditions, IR light exposure and non-exposure resulted in similar levels of lens opacity. Taken together, our results indicate that the avoidance of tear film dehydration is crucial for effective reduction of the incidence and severity of the formation of lens opacity during mouse eye experiments [3, 5].

The phenomenon of reversible lens opacification in mice has been published by many researchers [1–4, 14]. In our study, reversion of lens opacity spontaneously occurred after 6 h in both anaesthetised mice and IR light-exposed mice which did not receive anaesthetic during experiments. In another study, lens opacity induced by high NaCl osmolarity showed complete reversion at 90 min after application of a lower osmolarity solution for 1 h [6]. Interestingly, the authors of that study reported that naturally induced lens opacity was completely reversed in 30 min, which was equal to the time required for development of lens opacity in male C57BL/6 J mice (16–18 weeks of age) [6]. Thus, with the application of non-preserved artificial tears in anaesthetised mice, the time required for the reversion of lens opacity might decrease. In our study, we used non-preserved artificial tears to lubricate the ocular surface of anaesthetised mice every 1 min. Further studies are ongoing to evaluate the effect of different kinds of artificial tears, along with various states of osmolarity, on ocular surface lubrication and the formation of lens opacity.

A previous study reported that the formation of lens opacity significantly increased with lower body temperature, concluding that exposure to ≤23 °C caused cataracts in wild-type SV129 male mice [1]. However, Ridder et al. reported that a small temperature difference was not the primary source of lens opacity [3]. In our study, during the course of the experiment, the mice were placed on a heating pad set at 37 °C to prevent their body temperatures from falling below 23 °C. Although the formation of lens opacity is more likely related to ocular surface dryness than to body temperature, maintenance of body temperature may produce consistent results in anaesthetised mice [1].

Furthermore, there may be an effect of topical phenylephrine and tropicamide in the formation of lens opacity. Mydriatic drugs might decrease aqueous secretion, thereby affecting ocular osmolarity and fluid homeostasis [15]. In our study, to minimise the effect of topical phenylephrine and tropicamide, we applied one drop for all mice, immediately after anaesthesia.

We developed a modified lens opacity classification system to assess the severity of lens opacity in greater detail. Compared with the simple classification system by Bermudez et al. (numerical values from 0 to 3), our system uses six grades of lens opacity (Fig. 2) [1]. Because OCT devices are increasingly available for mouse eye research, we expect that our modified lens opacity classification system will be helpful for researchers in this field.

The methodological limitation of this study was its small sample size. To validate our results, a larger sample size for each group is warranted. Considering the importance of ocular surface hydration and ocular fluid homeostasis in the formation of lens opacity, further investigations of the relationships between ocular surface dryness and other confounding factors should be performed; moreover, analyses of changes in ocular fluid content and ion concentrations are needed. Because ketamine/xylazine anaesthesia in rats can produce acute hyperglycaemia, associations between lens opacity and a variety of metabolic changes after anaesthetisation with ketamine/xylazine also need to be explored [16].

## Conclusions

We demonstrated that ocular surface dryness might be a primary factor in the formation and progression of lens opacity in anaesthetised mice. This opacity was restored to normal status spontaneously after several hours. We believe that our results may enable researchers to minimise the formation of lens opacity during ocular phenotype studies in mutant mice. In addition, our data provide an important element of the protocol to maintain optical transparency during mouse eye experiments.

## Data Availability

The dataset supporting the conclusions of this article is included within the article and its additional file.
